# IFI35 is involved in the regulation of the radiosensitivity of colorectal cancer cells

**DOI:** 10.1186/s12935-021-01997-7

**Published:** 2021-06-03

**Authors:** Yan Hu, Bing Wang, Ke Yi, Qingjun Lei, Guanghui Wang, Xiaohui Xu

**Affiliations:** 1 Central Laboratory, The First People’s Hospital of Taicang, Taicang Affiliated Hospital of Soochow University, No. 58 Changsheng South Road, Taicang, 215400 Jiangsu China; 2 Department of General Surgery, The First People’s Hospital of Taicang, Taicang Affiliated Hospital of Soochow University, Taicang, Jiangsu China; 3 School of Pharmacy, Soochow University, Suzhou, Jiangsu China

**Keywords:** Colorectal cancer, IRF1, IFI35, Radiosensitivity, Luciferase reporter assay

## Abstract

**Background:**

Interferon regulatory factor-1 (IRF1) affects the proliferation of colorectal cancer (CRC). Recombinant interferon inducible protein 35 (IFI35) participates in immune regulation and cell proliferation. The aim of the study was to examine whether IRF1 affects the radiation sensitivity of CRC by regulating IFI35.

**Methods:**

CCL244 and SW480 cells were divided into five groups: blank control, IFI35 upregulation, IFI35 upregulation control, IFI35 downregulation, and IFI35 downregulation control. All groups were treated with X-rays (6 Gy). IFI35 activation by IRF1 was detected by luciferase reporter assay. The GEPIA database was used to examine IRF1 and IFI35 in CRC. The cells were characterized using CCK-8, EdU, cell cycle, clone formation, flow cytometry, reactive oxygen species (ROS), and mitochondrial membrane potential. Nude mouse animal models were used to detect the effect of IFI35 on CRC.

**Results:**

IRF1 can bind to the IFI35 promoter and promote the expression of IFI35. The expression consistency of IRF1 and IFI35 in CRC, according to GEPIA (R = 0.68, *p* < 0.0001). After irradiation, the upregulation of IFI35 inhibited cell proliferation and colony formation and promoted apoptosis and ROS, while IFI35 downregulation promoted proliferation and colony formation and reduced apoptosis, ROS, and mitochondrial membrane potential were also reduced. The in vivo experiments supported the in vitro ones, with smaller tumors and fewer liver metastases with IFI35 upregulation.

**Conclusions:**

IRF1 can promote IFI35 expression in CRC cells. IFI35 is involved in the regulation of radiosensitivity of CRC cells and might be a target for CRC radiosensitization.

## Background

Colorectal cancer (CRC) is a malignant tumor of the intestine originating from the colon or rectum [1]. It is the third most common malignant disease in the world and the second leading cause of cancer death [2]. In the past few decades, the incidence and mortality of CRC worldwide are steadily increasing [3, 4]. According to statistics from the American Cancer Society, there were 145,600 new cases of CRC and 51,020 deaths in the United States in 2019 [5]. Radical surgery combined with adjuvant radiotherapy is the main method for the treatment of CRC [6, 7]. Nevertheless, inherent and acquired radiation resistance of many patients with CRC can lead to treatment failure. Many genes, including EGFR, P53, Bcl-2, and NF-κB, are related to the radiosensitivity of various tumor cells, but the clinical application of these markers in radiotherapy is still controversial [8]. Therefore, in order to improve the effectiveness of radiotherapy for CRC, there is an urgent need for further research and discovery of potential targets related to radiation resistance and the development of new treatment strategies to improve the radiosensitivity of CRC.

Interferon regulatory factor-1 (IRF1) is a member of the IRF family, which is stimulated by interferon-γ and/or tumor necrosis factor (TNF)-α. It is expressed in lymphocytes, monocytes, tumor cells, and many other cell types (including intestinal epithelial cells) [9]. Yuan et al. [10] found that IFN-γ can transcriptionally regulate IRF1 to induce miR-29b expression, which can inhibit the invasion and metastasis of CRC by inhibiting the PI3K/Akt pathway. A positive feedback loop is formed between IRF1 and miR-29b, and this regulation can significantly increase the sensitivity of CRC to IFN-γ, thus providing a new way for the development and treatment of CRC.

Recombinant interferon inducible protein 35 (IFI35) (also known as IFP35) is a leucine zipper protein regulated by interferon. IFI35 is expressed in a variety of cell types, including monocytes/macrophages, epithelial cells, and fibroblasts [11]. Co-localization experiments with dual immunofluorescence and confocal laser scanning microscopy showed that IFI35 has no specific localization relationship with any organelles, including mitochondria, peroxisomes, endoplasmic reticulum, lysosomes, endosomes, Golgi complex, ribosomes, and actin, among others [12]. The subcellular structure typing of membrane-associated proteins isolated from cytoplasmic proteins indicates that IFI35 is localized in the cytoplasm [12]. Electron microscopy studies showed that IFI35 aggregated into cytoplasmic clusters in IFN-treated cells, and these cells were neither associated nor surrounded by a membrane [12]. Combining immunoprecipitation and immunofluorescence studies, cells transfected with the hemagglutinin epitope IFI35 expression construct showed complex formation and co-localization of endogenous and transfected IFI35 [12]. Nmi and IFI35 can form Nmi-IFI35 physical association. Interferon-alpha induces nmi-IFP35 heterodimeric complex formation that is affected by the phosphorylation of IFI35. Nmi could induce apoptosis; contrarily, IFI35 acts against the proapoptotic effect of Nmi when expressed together; and IFI35 promotes survival, inhibits cell death in some cancers [13, 14]. CKIP-1 could bind to Nmi and release IFI35, leading to IFI35 degradation. Consistently, CKIP-1 could inhibit tumor cell growth and inhibit Akt mediated cell survival. These results strongly suggest that CKIP-1, IFI35 and Nmi have biological relevance through interactions. Moreover, CKIP-1 has been implicated in the regulation of muscle differentiation, apoptosis, cell morphology and cytoskeleton [15]. In summary, IFI35 is one of interferon-inducible proteins, which is involved in the regulation of tumor cell functions such as cell survival and apoptosis. However, there are few similar reports in colorectal cancer.

A previous study by our group showed that the expression of the interferon-induced proteins IFI6, IFI35, and IFITM1 was increased after IRF1 was upregulated [16]. Other studies also showed that these three interferon-induced proteins are involved in the immune response caused by viral infections [17–19]. Nevertheless, they are also involved in the development and prognosis of various tumors. Cheriyath et al. [20] found that mtROS induced by IFI6 has a direct role in the formation of migration structures and nuclear gene expression, which can promote breast cancer cell metastasis, and interrupting the mitochondrial function of IFI6 may improve the prognosis of breast cancer. In addition, both IRF1 and IFI6 are positively correlated with the drug-specific chemical sensitivity of gastric cancer [21]. Lui et al. [22] found that the over-expression of IFITM1 could promote the occurrence and development of breast cancer, and targeted therapy against IFITM1 might be beneficial to breast cancer patients who are resistant to endocrine therapy. IFI35 negatively regulates IFN-β phosphorylation of the STAT1-RIG-I-CXCL10/CCL5 axis in polyinosinic acid-polycytidylic acid-treated astrocytoma cells [23]. Yang et al. [24] found that IRF1 could directly bind to a site of the IFI35 promoter and activate IFI35 expression in HeLa cells in an IFN-γ-induced manner. In view of those results and our previous results, IFI35 was selected as the subject of this research.

In view of the special structure of IFI35 and characteristics in cells, and since IFI35 is involved in the immune regulation of various diseases and the apoptosis of tumor cells [14], it could be hypothesized that IFI35 and IRF1 downstream could participate in the radiosensitivity of CRC. Therefore, the aim of the study was to examine whether IRF1 affects the radiation sensitivity of CRC by regulating IFI35. The results could provide additional data for eventually circumventing radiation resistance in cancer.

## Methods

### Cell culture

Our previous study revealed that CCL244 and SW480 cells were relatively radiation-resistant cell lines among seven types of CRC cells [25]. The present study mainly explored how to enhance the radiation sensitivity of CRC. Therefore, these two types of cells were used for in vitro experiments. CCL244 and SW480 cells were obtained from the Chinese Academy of Sciences Cell Bank (Shanghai, China) and maintained in Dulbecco’s modified Eagle’s medium (Hyclone, Thermo Fisher Scientific, Waltham, MA, USA) supplemented with 10% fetal bovine serum and 100 U/mL penicillin-streptomycin at 37 ℃ and 5% CO_2_.

### IRF1 and IFI35 infection

The shRNA against IFI35 (5’-GCT CAA CAT TCC TGA TAT CTT TCA AGA GAA GAT ATC AGG AAT GTT GAG CTT TTT T-3’), IRF1 (sh1: 5’-GGC TCA TCT GGA TTA ATA AAG TTC AAG AGA CTT TAT TAA TCC AGA TGA GCC TTT TTT-3’, sh2: 5’-GGA AAT TAC CTG AGG ACA TCA TTC AAG AGA TGA TGT CCT CAG GTA ATT TCC TTT TTT-3’) and NC as control (5’-TTC TCC GAA CGT GTC ACG TTT CAA GAG AAC GTG ACA CGT TCG GAG AAT TTT TT-3’) were obtained from Vigene Biosciences (Vigene Biosciences, Inc., Rockville, MD, USA), inserted into the lentivirus (LV) expression vectors and packaged into viral particles. CCL244 and SW480 cells were used for subsequent infection. After 72 h, the cells were harvested and selected in a medium containing 3 µg/mL puromycin (Sigma-Aldrich, St. Louis, MO, USA). The stable IFI35 or IRF1 knocked-down cell lines were validated using Western blot.

For viral infection, the adenovirus expression vectors IFI35 (Ad-IFI35), IRF1 (Ad-IRF1), and negative control (Ad-NC) were obtained from Hanbio Biotechnology (containing RFP, Shanghai, China). The cells were infected by Ad-IFI35, Ad-IRF1, and Ad-NC to achieve IRF1 and IFI35 overexpression and used for the relevant experiments 48 h after infection.

### Detection of luciferase reporter gene

The pGL-3 vector containing luciferase IFI35 promoter, blank control pGL-3 basic, and Renilla luciferase reporter gene vector pRL-TK-Renilla were from Jinan Weizhen Co. (Jinan, China). The Ad-IRF1 and Ad-NC cells were digested and placed in 24-well plates. After the cells adhered, pGL-3 basic, pGL-3-IFI35, and TK were transfected using Lipofectamine 3000 (Invitrogen Inc., Carlsbad, CA, USA) according to the manufacturer’s instructions. After transfection for 6 h, the medium was replaced. The cells were incubated at 37 °C for 48 h. The medium was removed, and the cells were washed with 100 µL of PBS twice. The 1× Passive Lysis Buffer (Dual-Luciferase Reporter Assay System E1910, Promega, Madison, WI, USA) was prepared and added (200 µL) to each well. After 20 min, the mixture was centrifuged at 13,000 rpm for 30 s, and 50 µL of the supernatant was transferred to a white opaque 96-well microplate. The pre-mixed LARII solution (30 µL) of the Promega Dual-Luciferase kit (Dual-Luciferase Reporter Assay System E1910, Promega, Madison, WI, USA) to each well. The results were read on a microplate reader (Varioskan LUX, Thermo Fisher Scientific, Waltham, MA, USA). Finally, a BCA kit (P0011, Beyotime, Shanghai, China) was used to determine the protein concentration of each well for correction.

### GEPIA database

The results of IRF1 and IFI35 in CRC were validated using the GEPIA database, which is a database for cancer and normal gene expression [26].

### Cell proliferation assay

The logarithmic-phase cells of the IFI35 groups were resuspended to 1 × 10^4^/well in 100 µL in 96-well plates, with eight replicate wells were each set. After adherence, the cells were irradiated with 6 Gy of X-rays. Cell viability was measured at 24, 48, and 72 h after irradiation using a CCK-8 kit (PF724, Dojindo Molecular Technologies, Kimamoto, Japan). A multi-functional microplate reader was used to detect the OD value at a wavelength of 450 nm. The cell viability rate was calculated with the following formula: cell viability rate = (OD in experimental group/OD in the mock group) × 100%. Graphpad 6.0 software (GraphPad Software Inc., San Diego, CA, USA) was used to analyze and plot the growth curve. Three independent experiments were performed in quadruplicates.

### Colony formation assay

The cells were seeded on 6-well plates at a density of 2000 cells/well. After 24 h, both CCL244 and SW480 were exposed to X-rays with a dose of 6 Gy. After 14 days of incubation at 37 °C, the colonies were stained with Giemsa (418,033, Besso Biotechnology Co., Ltd., Zhuhai, China), and those with a minimum of 50 viable cells were counted. This process was repeated three times. The cloning efficiency was calculated as a ratio of the number of colonies formed divided by the total number of cells plated.

### Flow cytometric analysis of cell apoptosis assays

Following conventional digestion, cells in the logarithmic growth phase were used to prepare a single cell suspension; 2 × 10^5^ cells/mL were seeded in 6-well plates. After treatment for 48 h, all cells were collected and centrifuged at 2000 rpm for 5 min; the supernatant was discarded after washing with PBS, and centrifugation was repeated twice. The cells were stained with fluorescein FITC-conjugated Annexin V and PI (40302ES60, Yeasen, Shanghai, China) and analyzed by flow cytometry (Beckman Coulter, Brea, CA, USA). The ratio of both early and late apoptosis cells was calculated [25].

### Flow cytometric analysis of cell cycle

After treatment, cells in the logarithmic growth phase were collected and fixed with 70% precooled ethanol overnight. After staining with propidium iodide (10 µg/mL, Sigma-Aldrich, St Louis, MO, USA) in the dark for 30 min, flow cytometry was performed on a FACSCalibur Flow Cytometer system (BD Biosciences, San Jose, California, USA) and the cell cycle distribution was analyzed with the Flow-Jo 7.6 software (BD Biosciences, San Jose, California, USA) [27].

### Western blotting

The proteins in cell lysates were resolved using sodium dodecyl sulfate-polyacrylamide gel electrophoresis and transferred to a nitrocellulose membrane, which was blocked with PBS/Tween-20 containing 5% bovine serum albumin. The membranes were incubated with antibodies against IRF1 (1:500, ab186384, Abcam, Cambridge, UK), IFI35 (1:500, ab233415, Abcam, Cambridge, UK), Bcl-2 (1:1000, #15,071, Cell Signaling Technology, MA, USA), cleaved Caspase-3 (1:500, ab2302, Abcam, Cambridge, UK), cleaved Caspase-9 (1:200, ab2324, Abcam, Cambridge, UK), Bax (1:1000, #5023, Cell Signaling Technology, Inc., Danvers, MA, USA), and Tubulin (1:1000, GB13017-2, Servicebio, Woburn, MA, USA). Goat anti-mouse primary antibodies (1:1000, A0208) and goat anti-rabbit secondary antibodies (1:1000, A0216) were from Beyotime (Shanghai, China).

### Measurement of mitochondrial membrane potential

CRC cells of different treatment groups were inoculated into a six-well plate at a cell density of 60%. After the cells adhered, there were irradiated with 6 Gy of X-rays. After 24 h, the medium was removed, and the cells were washed three times with PBS. Then, 500 µL of JC-1 probe (1:1000, ab113850, Abcam, Cambridge, UK) diluted in serum-free medium (DMEM and RPMI1640) was added to each well. The cells were incubated at 37 °C for 1 h. The medium was removed, the cells were washed three times with PBS, and the cells were imaged at 20× under a microscope (DM IL LED, Leica, Wetzlar, Germany).

### Detection of oxygen free radicals

The cells were resuspended in a six-well plate. After the cells adhered, they were irradiated with 6 Gy of X-rays. After 12 h, the medium was discarded, and the cells were washed three times with PBS. After digesting with trypsin, the cells were resuspended, placed in a centrifuge tube, and centrifuged at 1200 rpm for 5 min. The cells were washed twice with PBS. After centrifugation, the supernatant was discarded, and the cell pellet was collected. The fluorescent probe DCFH-DA (S0033M, Beyotime, Shanghai, China) was diluted 1:1000 with PBS, and the mixture was added to resuspend the cell pellet and placed in a 37 °C water bath for 30 min. The cells were shaken every 5 min. After loading the fluorescent probe, the mixture was centrifuged at 1200 rpm for 5 min; the cells were washed three times with PBS, resuspended in 1 mL of new PBS, and, finally, flow cytometry was used for detection.

### Animal experiments

Four-week-old male BALB/c nude mice were purchased from Shanghai SLAC Laboratory Animal Co., Ltd. (Shanghai, China). The mice were maintained under standard laboratory conditions on a 12-h light-dark cycle and given access to sterilized food and water under a specific pathogen-free environment. For the subcutaneous injection, CCL244 cells (5 × 10^4^) were suspended in 100 µL of PBS and inoculated subcutaneously into the right posterior flank region of the mice. The mice were divided into five groups: (1) Control group without treatment; (2) Ad-IFI35 group; (3) Ad-NC group; (4) shRNA-IFI35 group; and (5) shRNA-NC group (n = 5/group). Two-dimensional measurements were taken with an electronic caliper every 3 days, and the tumor volume in mm^3^ was calculated using the formula: volume = a × b^2^/2, where a was the longest diameter and b the shortest diameter. When the tumor volume in all groups reached 80 mm^3^ on the fifth day, the local tumor was irradiated with 8 Gy of X-ray. Tumor volume was measured every 4 days, and the mice were sacrificed on the 29th day. The tumors were frozen at **− **80 °C or fixed in 10% formalin overnight and subjected to routine histological examination. Next, liver metastasis models were established by tail vein injection of IFI35-up/down-regulated and control CCL244 cells (the grouping is the same as above). The metastatic liver tumors were frozen at − 80 °C or fixed in 10% formalin overnight and subjected to routine histological examination. The method of sacrifice of the animals was as follows: The nude mouse was placed into the euthanasia box, and carbon dioxide was infused into the box at a rate of 10–30% of the solvent in the euthanasia box per min. It was ensured that the nude mouse did not move, had no breathing and had dilated pupils. The carbon dioxide was turned off, followed by observation for 2 min to confirm that the nude mouse was dead.

### Ethics

This study was approved by the Institutional Animal Care and Use Committee (#KY-2020-010) [28–30].

### Hematoxylin and eosin (H&E) staining

Subcutaneous tumor and liver tissues were fixed in 10% neutral-buffered formalin and embedded in paraffin. Paraffin sections (about 3 μm) were deparaffinized and heat-treated with citrate buffer, pH 6.0, for 7 min following an epitope retrieval protocol. The sections of the subcutaneous tumor and liver were stained with H&E. All slides were examined under a microscope and photographed. Representative images were randomly selected from each sample.

### Immunohistochemistry

Section (3 μm) were deparaffinized, and the antigens were retrieved in citrate buffer (pH 6.0) under heat. Endogenous peroxidase was blocked using H_2_O_2_ for 15 min, and non-specific binding was blocked with 4% skim milk for 30 min. The sections were incubated with the primary antibody against Bcl-2 (1:100, Abcam, Cambridge, UK) overnight at 4 °C. The secondary antibody was added and incubated 30 min at room temperature and revealed using the Dako LSAB2 system (Dako, Glostrup, Denmark). The sections were counterstained with hematoxylin. The sections were scored as negative (− , < 10% of staining), weakly positive (+, 10− 25% of staining), moderately positive (++, 25− 75% of staining), and strongly positive (+++, > 75% of staining).

### Statistical analysis

Data are expressed as means ± standard deviation. The data were evaluated using an unpaired two-sided Student’s t-test after confirming that the data met appropriate assumptions (normality, homogeneous variance, and independent sampling). When more than two groups were compared, one-way ANOVA was adopted followed by Tukey’s post hoc test. For all in vitro experiments, five biological replicates were analyzed. For all in vivo experiments, three biological replicates were analyzed for each condition. Statistical analysis was performed using Prism 6.01 software (GraphPad Software, La Jolla, CA, USA). The differences were considered significant if *p* < 0.05 (*), *p* < 0.01 (**), *p* < 0.001 (***), and *p* < 0.0001 (****).

## Results

### In CRC cells, IRF1 can bind to the IFI35 promoter and promote IFI35 expression, and the two genes are positively correlated in CRC

**Fig. 1:**
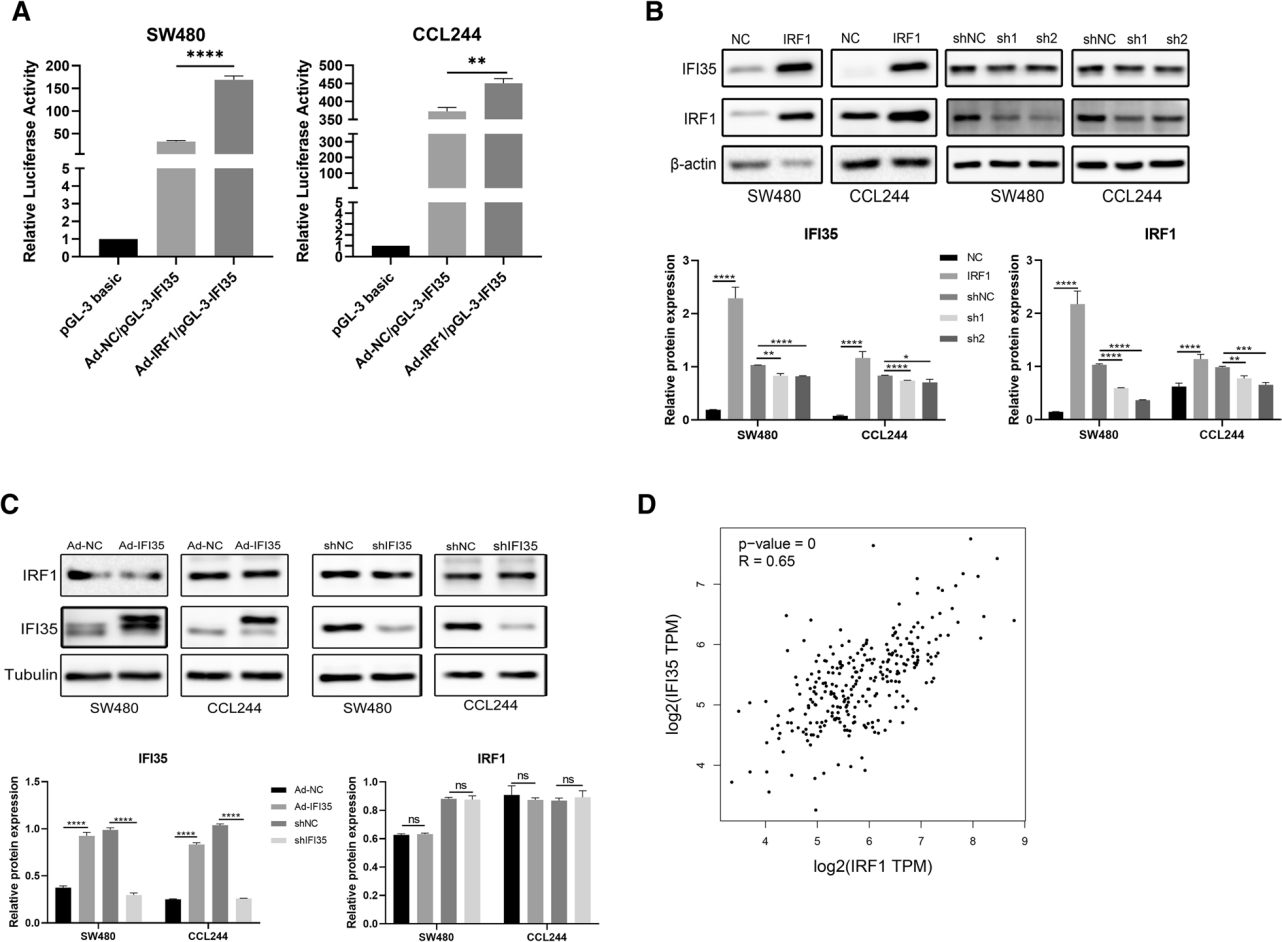
IRF1 regulates the expression of IFI35, and their expression is positively correlated. **A** Luciferase reporter assay for the binding of IRF1 and IFI35 promoter and the regulation of IRF1 on IFI35. The bars show the fold of IFI35 expression relative to the empty vector. **B** Western blot was used to verify the expression of IFI35 after down- or upregulating IRF1. The results suggest that IRF1 modulates the expression of IFI35. **C** Western blot was used to verify the expression of IRF1 after down- or upregulating IFI35. The results suggest that IFI35 does not modulate the expression of IRF1. **D** The correlation between IRF1 and IFI35 expression was retrieved from the GEPIA database. Ad-NC: adenovirus, negative control; Ad-IRF1, adenovirus for IRF1 overexpression; sh: short hairpin RNA for the downregulation of IRF1 or IFI35. shNC: negative control short hairpin RNA

The luminescence values were detected in three groups: TK + pGL-3 basic, Ad-NC:TK + pGL-3-IFI35, and Ad-IRF1:TK + pGL-3-IFI35. The results showed that compared with the control group, the expression of the luciferase reporter gene in the promoter region containing IFI35 was significantly increased after IRF1 was upregulated, suggesting that IRF1 could bind to and activate the promoter region of IFI35 and promote the expression of IFI35 (Fig. 1A). After infecting CRC cells with downregulating lentivirus and upregulating adenovirus, Western blot was used to detect the expression of the IRF1 and IFI35 proteins in CRC cells. It was found that IRF1 could affect IFI35 expression (Fig. 1B), but that IFI35 could not affect the expression of IRF1 in turn (Fig. 1C). By searching the GEPIA database, it was found that the expression of IRF1 and IFI35 in CRC had a strong consistency (R = 0.68, *p* < 0.0001) (Fig. 1D). Taken together, the results suggest that IRF1 can regulate the expression of IFI35, and their expression is positively correlated.

### IFI35 upregulation combined with X-ray can significantly inhibit the proliferation and colony formation of CRC cells

**Fig. 2:**
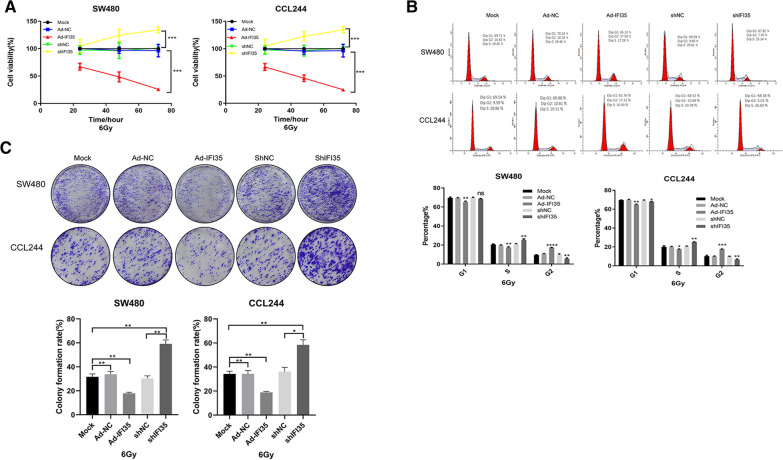
Effect of IFI35 on X-ray irradiation and the proliferation and colony formation of colorectal cancer cells. **A** CCK8 was used to detect the effect of IFI35 on the proliferation of colorectal cancer cells. Overexpression of IFI35 decreases colorectal cancer cell viability, while the inhibition of IFI35 increases their viability. **B** Flow cytometry detection of the effect of IFI35 on the cell cycle of colorectal cancer cells. The bar graph shows the proportion of cells in each cell cycle phase. Overexpressing IFI35 increases the proportion of cells in G2, while inhibiting IFI35 decreases the proportion of cells in G2. **C** The effect of IFI35 on the colony formation rate of colorectal cancer cells was verified by the colony formation test. The bar graph shows the colony formation rates. Overexpression IFI35 decreases the colony formation, while inhibiting IFI35 increase colony formation. *p < 0.05, **p < 0.01, ***p < 0.001 vs. Ad-NC or shNC, as appropriate. Ad-NC: adenovirus, negative control; Ad-IRF1, adenovirus for IRF1 overexpression; sh: short hairpin RNA for the downregulation of IRF1 or IFI35. shNC: negative control short hairpin RNA

CCK8 was used to detect the proliferation of CRC cells. It was found that the upregulation of IFI35 could significantly inhibit the proliferation of CRC cells, whereas the downregulation of IFI35 promoted the proliferation of CRC cells (Fig. 2A). Flow cytometry was used to detect the cell cycle changes of CRC cells. It was found that the upregulation of IFI35 could significantly prolong the G2 phase, while the G1 and the S phases were relatively reduced. On the other hand, the downregulation of FI35 could cause the G2 phase to shorten significantly, and the G1 and S phases increased relatively. IFI35 could significantly inhibit the division of colorectal cancer cells, leading to a delay of cell entry into the G1 and S phases, thus leading to G2 phase arrest and shortening of the G1 and S phases (Fig. 2B). Cells of different treatment groups were inoculated in 60-mm cell culture dishes. Each group of cells was seeded with 2000 cells. After the cells adhered, they were fixed 14 days after irradiation, stained with Giemsa, and counted. The results showed that the clone formation rate after IFI35 was upregulated was significantly lower than that of the other groups, while the clone formation rate was significantly increased after IFI35 was downregulated (Fig. 2C). Taken together, X-ray irradiation after the upregulation of IFI35 could significantly inhibit the proliferation and colony formation of CRC cells and increase the radiation sensitivity of CRC cells.

### IFI35 upregulation combined with X-ray can obviously promote the apoptosis of CRC cells and promote the expression of pro-apoptotic proteins

**Fig. 3:**
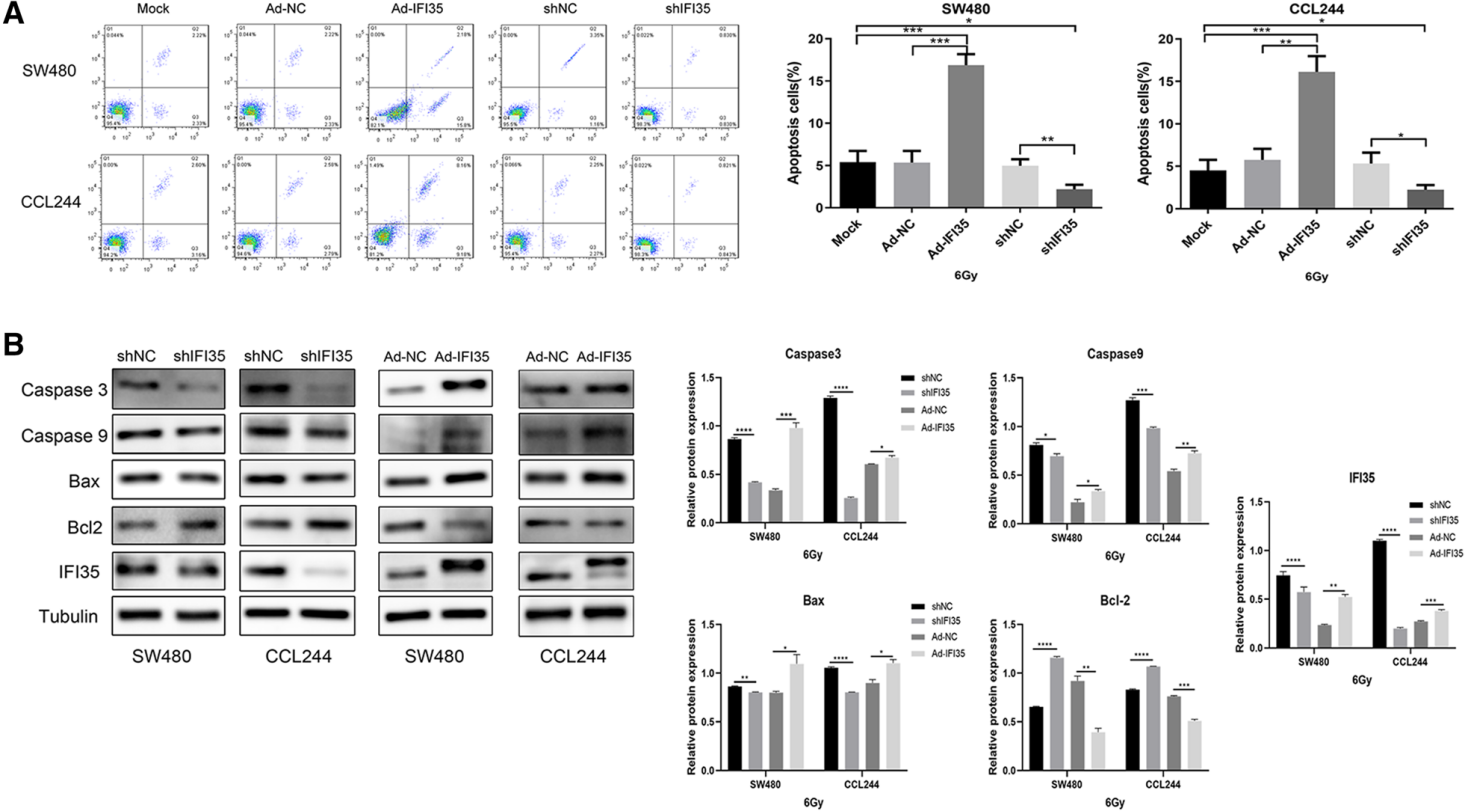
Effect of IFI35 on X-ray irradiation and cell apoptosis of colorectal cancer cells. **A** Flow cytometry detection of the effect of IFI35 on the cell apoptosis of colorectal cancer cells. The bar graph shows the proportion of apoptotic cells. Overexpressing IFI35 increases cancer cell apoptosis, wile inhibiting IFI35 decreases cancer cell apoptosis. **B** Expression of apoptosis-related proteins Bax, Caspase-3, Caspase-9, and Bcl-2 determined by Western blot. The bar graphs show the protein expression normalized to that of GAPDH. *p < 0.05, **p < 0.01, ***p < 0.001, ****p < 0.0001. Ad-NC: adenovirus, negative control; Ad-IRF1, adenovirus for IRF1 overexpression; sh: short hairpin RNA for the downregulation of IRF1 or IFI35. shNC: negative control short hairpin RNA

Flow cytometry was used to detect the changes in CRC cell apoptosis in different treatment groups. It was found that the upregulation of IFI35 could obviously promote the apoptosis of CRC cells caused by irradiation, while the downregulation of IFI35 could significantly reduce the apoptosis caused by irradiation. Flow cytometry was used to detect the changes in CRC cell apoptosis in the different treatment groups. It was found that the upregulation of IFI35 could obviously promote the apoptosis of CRC cells caused by irradiation, while the downregulation of IFI35 could significantly reduce the apoptosis caused by irradiation (Fig. 3A). In addition, Western blot was used to detect the expression of apoptosis-related proteins. It was found that the upregulation of IFI35 could significantly promote the expression of Bax, caspase-3, and caspase-9 and inhibit the expression of Bcl-2. On the other hand, downregulating IFI35 inhibited Bax, caspase-3, and caspase-9 expression and promoted Bcl-2 expression (Fig. 3B). In summary, IFI35 could significantly increase the apoptosis of CRC cells caused by X-rays.

### IFI35 upregulation combined with X-ray could significantly promote reactive oxygen species and increase mitochondrial membrane potential in CRC cells

**Fig. 4:**
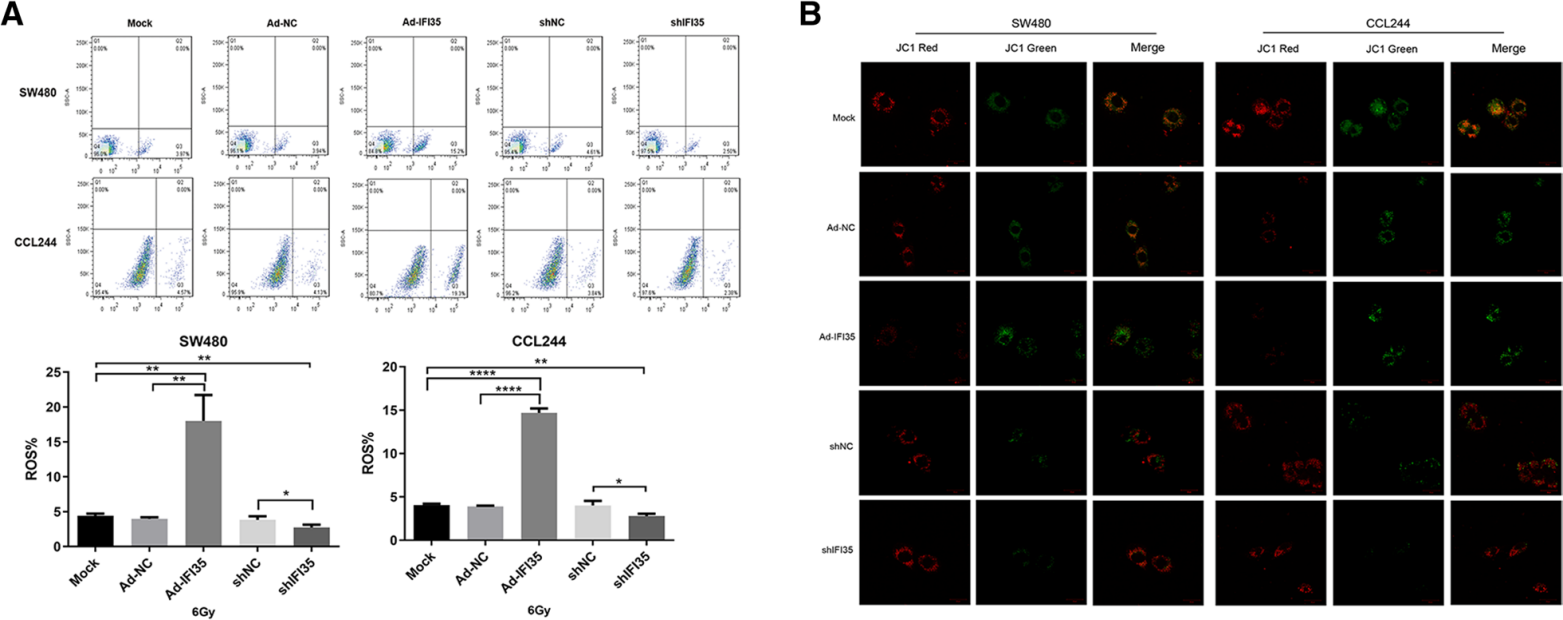
Effect of IFI35 on X-ray irradiation and reactive oxygen species and mitochondrial membrane potential of colorectal cancer cells. **A** Flow cytometry detection of the effect of IFI35 on reactive oxygen species (ROS) of colorectal cancer cells. The bar graphs show the production of ROS. *p < 0.05, **p < 0.01, ***p < 0.001, ****p < 0.0001. **B** Mitochondrial membrane potential of colorectal cancer cells determined by JC-1 immunofluorescence. Ad-NC: adenovirus, negative control; Ad-IRF1, adenovirus for IRF1 overexpression; sh: short hairpin RNA for the downregulation of IRF1 or IFI35. shNC: negative control short hairpin RNA

At 48 h after irradiation, flow cytometry was used to detect oxygen free radicals in the different treatment groups. The results showed that the oxygen free radical content significantly increased after IFI35 was upregulated, and the oxygen free radical content was significantly reduced after IFI35 was downregulated (Fig. 4A). Therefore, it indirectly reflects that the upregulation of IFI35 can lead to increased DNA damage of CRC cells, and the downregulation of IFI35 can reduce the DNA damage of cells.

The different treatment groups were stained with JC-1 after being irradiated with 6 Gy for 24 h. The results showed that compared with the blank control and the negative control groups, the green fluorescence increased, and the mitochondrial membrane potential increased after IFI35 was upregulated. After IFI35 was downregulated, JC-1 resumed aggregation in the mitochondrial matrix and produced red fluorescence, and the green fluorescence was weaker (Fig. 4B). Therefore, the results show that the upregulation of IFI35 could significantly increase the radiation-induced increase in mitochondrial membrane potential of CRC cells and induce apoptosis.

### IFI35 upregulation combined with X-ray could significantly inhibit the formation of CRC subcutaneous tumors and distant metastasis

**Fig. 5:**
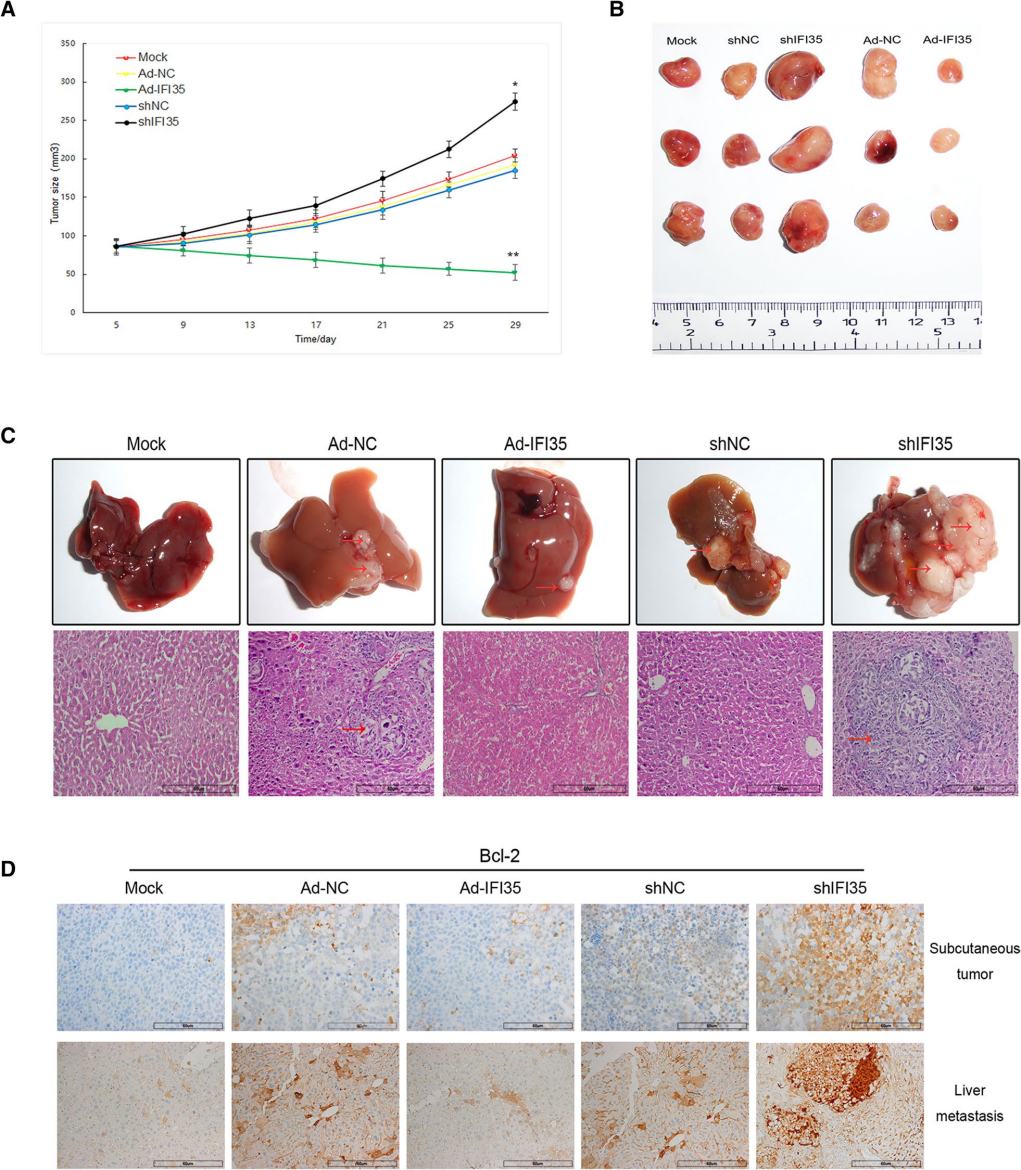
In vivo experiments supporting the effect of colorectal cancer on X-ray radiosensitivity after IFI35. Four-week-old BALB/c nude mice were divided into five groups: (1) Control group without treatment; (2) Ad-IFI35 group; (3) Ad-NC group; (4) shRNA-IFI35 group; and (5) shRNA-NC group (n = 5/group). **A** Tumor volume growth curve of each group. Two-dimensional measurements were taken with an electronic caliper every 3 days, and the tumor volume in mm^3^ was calculated. **B** Representative subcutaneous tumors of each group. **C** Liver metastasis models were established by tail vein injection of IFI35-up/down-regulated and control CCL244 cells (grouping was the same as above). Liver metastasis specimens of colorectal cancer and HE staining of liver metastases in each group. The red arrows point at metastatic lesions in the specimen and the HE staining. **D** Immunohistochemistry was used to detect the expression of the apoptotic protein Bcl-2 in subcutaneous tumors and liver metastases. Ad-NC: adenovirus, negative control; Ad-IRF1, adenovirus for IRF1 overexpression; sh: short hairpin RNA for the downregulation of IRF1 or IFI35. shNC: negative control short hairpin RNA

After the X-ray irradiation of CRC cells, the in vivo experiments showed that the growth rate of subcutaneous CRC was significantly inhibited after IFI35 was upregulated, while the growth rate of nude mice tumors after IFI35 downregulation was significantly higher than that of the other treatment groups (Fig. 5A). The tumors were the smallest in the IFI35 upregulation group and the largest in the IFI35 downregulation group (Fig. 5B). Under the condition of X-ray irradiation, the incidence of CRC liver metastasis was significantly reduced after IFI35 was upregulated, and the number of metastases and the size of metastases were lower than in the other groups. The incidence of liver metastases after IFI35 was downregulated was significantly higher than in the other treatment groups, and the number of metastasis and size of tumors was the highest. There were significant differences in liver tumor lesions among the different treatment groups, and the results were consistent with the general specimens (Fig. 5C). Immunohistochemistry was used to detect the apoptotic protein BCL-2 in CRC subcutaneous tumors and liver metastases. The results showed that irrespective of the lesion location, the expression of Bcl-2 was reduced after IFI35 was upregulated, but the expression of Bcl-2 was significantly increased in IFI35 downregulated tissues, which means that IFI35 could obviously promote the apoptosis of CRC cells in vivo (Fig. 5D). These results are consistent with the results of the cell experiments.

## Discussion

IRF1 affects the proliferation of CRC [10]. Consistent with this, our previous study found that IRF1 is involved in the progression of CRC [16]. IFI35 participates in immune regulation and cell proliferation. What‘s more, we found that IRF1 can positively regulate the expression of IFI35 in CRC cells, as analyzed by RNA sequencing and western blotting [16]. We also got the same result when searching GEPIA database, that is expression of IRF1 and IFI35 in CRC had a strong consistency (Fig. 1D). The role of IRF1 and IFI35 in CRC radiosensitivity is poorly understood. Therefore, the aim of the study was to examine whether IRF1 affects the radiation sensitivity of CRC by regulating IFI35. The results suggest that IRF1 can promote IFI35 expression by binding to the IFI35 promoter in CRC cells. IFI35 is involved in the regulation of radiosensitivity of CRC cells and might be a target for CRC radiosensitization.

**Fig. 6:**
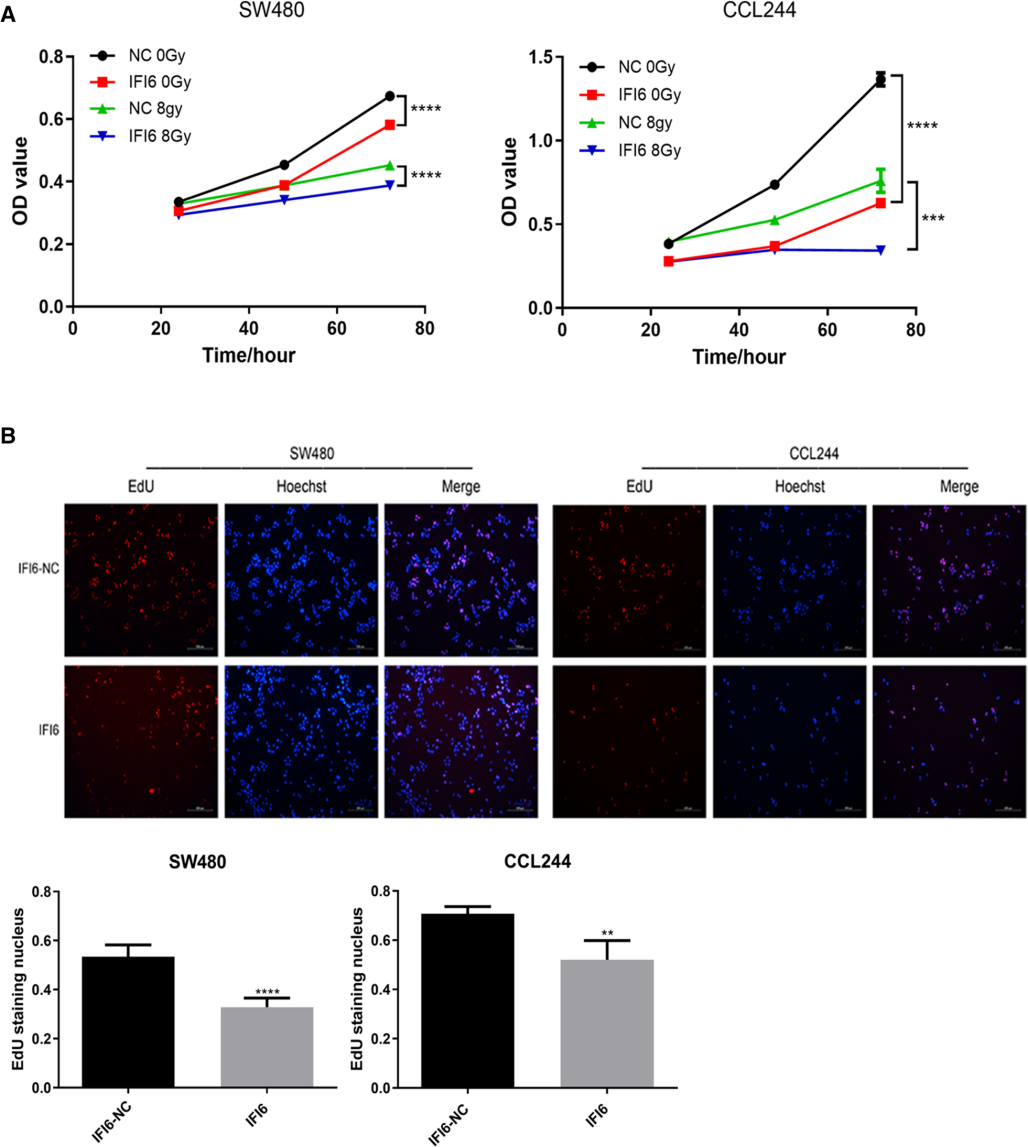
**A** CCK8 was used to detect the effect of IFI6 on the proliferation of colorectal cancer cells. **B** EdU was used to detect the effect of IFI6 on the proliferation of colorectal cancer cells. ***p < 0.001, ****p < 0.0001. NC: negative control

In this study, after X-ray irradiation, the upregulation of IFI35 could significantly inhibit the proliferation and colony formation of CRC cells. In addition, G2 phase arrest occurred, and the production of oxygen free radicals, mitochondrial membrane potential, and the apoptosis rate increased significantly. On the other hand, the reverse results were obtained after IFI35 was downregulated. These experimental results indicate that the upregulation of IFI35 could significantly promote the radiosensitivity of CRC cells. This phenomenon might be mainly achieved by regulating the oxygen free radicals of CRC cells and the mitochondrial membrane potential to affect apoptosis. In addition, consistent results were obtained with the animal experiments and showed that the upregulation of IFI35 could significantly inhibit liver metastasis of CRC, which is also consistent with the results caused by the upregulation of IRF1 reported earlier [16]. Therefore, the effect of IFN-γ-mediated IRF1 on the occurrence and development of colorectal cancer cells and radiosensitivity might be achieved by regulating IFI35. Interestingly, we chose another interferon-inducible protein IFI6 as a comparison and found that the proliferation of CRC cells was also significantly inhibited after IFI6 was upregulated (Fig. 6). This result implies that IRF1 might play a role by regulating a series of interferon-induced proteins in the occurrence and development of CRC and the radiosensitivity of CRC, but it is still not clear whether IRF1 can directly participate in the regulation of IFI6 promoter and the expression of IFI6. Nevertheless, it is clear that IRF1 can directly participate in the regulation and expression of IFI35 and form the IFN-γ/IRF1/IFI35 axis to play a role in CRC. Indeed, IFI6 has a direct role in the formation of migration structures in breast cancer metastasis [20]. In addition, the expression of IRF1 and IFI6 are is associated with drug sensitivity in gastric cancer [21]. IFITM1 promotes breast cancer [22]. IFI35 negatively regulates IFN-β phosphorylation in astrocytoma cells [23]. IRF1 directly binds to the IFI35 promoter and activates IFI35 expression in HeLa cells in an IFN-γ-induced manner [24]. Nevertheless, the exact mechanisms involved remain to be elucidated.

## Conclusions

In conclusion, IFI35 is regulated by IRF1 and is involved in the proliferation, metastasis, and radiation sensitivity of CRC. IRF1/IFI35 is not the only mechanism regulating the biological behavior of CRC, but it certainly plays an important role. The IRF1/IFI35 axis could be a treatment target against CRC.

## Data Availability

All data generated or analyzed during this study are included in this published article.

## Abbreviations

IRF1: Interferon regulatory factor-1
CRC: Colorectal cancer
IFI35: Recombinant interferon inducible protein 35
ROS: Reactive oxygen species
TNF: Tumor necrosis factor
H&E: Hematoxylin and eosin
CKIP-1: Casein kinase 2 interaction protein 1

## References

[1] Kuipers EJ, Grady WM, Lieberman D, Seufferlein T, Sung JJ, Boelens PG, van de Velde CJ, Watanabe T (2015). Colorectal cancer. Nat Rev Dis Primers.

[2] Sung H, Ferlay J, Siegel RL (2021). Global Cancer Statistics 2020: GLOBOCAN estimates of incidence and mortality worldwide for 36 cancers in 185 countries. CA Cancer J Clin..

[3] Wolf AMD, Fontham ETH, Church TR, Flowers CR, Guerra CE, LaMonte SJ, Etzioni R, McKenna MT, Oeffinger KC, Shih YT (2018). Colorectal cancer screening for average-risk adults: 2018 guideline update from the American Cancer Society. CA Cancer J Clin.

[4] Bailey CE, Hu CY, You YN, Bednarski BK, Rodriguez-Bigas MA, Skibber JM, Cantor SB, Chang GJ (2015). Increasing disparities in the age-related incidences of colon and rectal cancers in the United States, 1975–2010. JAMA Surg.

[5] Siegel RL, Miller KD (2019). Cancer statistics, 2019. CA Cancer J Clin..

[6] NCCN Clinical (2020). Practice Guidelines in Oncology (NCCN Guidelines). Colon Cancer. Version 4.2020.

[7] NCCN Clinical (2020). Practice Guidelines in Oncology (NCCN Guidelines). Rectal Cancer. Version 6.2020.

[8] Zheng S, Zhong YF, Tan DM, Xu Y, Chen HX, Wang D (2019). miR-183-5p enhances the radioresistance of colorectal cancer by directly targeting ATG5. J Biosci..

[9] Mannick EE, Cote RL, Schurr JR, Krowicka HS, Sloop GD, Zapata-Velandia A, Correa H, Ruiz B, Horswell R, Lentz JJ (2005). Altered phenotype of dextran sulfate sodium colitis in interferon regulatory factor-1 knock-out mice. J Gastroenterol Hepatol.

[10] Yuan L, Zhou C, Lu Y, Hong M, Zhang Z, Zhang Z, Chang Y, Zhang C, Li X (2015). IFN-γ-mediated IRF1/miR-29b feedback loop suppresses colorectal cancer cell growth and metastasis by repressing IGF1. Cancer Lett..

[11] Bange FC, Vogel U, Flohr T, Kiekenbeck M, Denecke B, Bottger EC (1994). IFP 35 is an interferon-induced leucine zipper protein that undergoes interferon-regulated cellular redistribution. J Biol Chem.

[12] Kerr CH, Skinnider MA, Andrews DDT, Madero AM, Chan QWT, Stacey RG, Stoynov N, Jan E, Foster LJ (2020). Dynamic rewiring of the human interactome by interferon signaling. Genome Biol.

[13] Zhou X, Liao J, Meyerdierks A, Feng L, Naumovski L, Bottger EC, Omary MB (2000). Interferon-alpha induces nmi-IFP35 heterodimeric complex formation that is affected by the phosphorylation of IFP35. J Biol Chem.

[14] Chen J, Naumovski L (2002). Intracellular redistribution of interferon-inducible proteins Nmi and IFP 35 in apoptotic cells. J Interferon Cytokine Res.

[15] Zhang L, Tang Y, Tie Y, Tian C, Wang J, Dong Y, Sun Z, He F (2007). The PH domain containing protein CKIP-1 binds to IFP35 and Nmi and is involved in cytokine signaling. Cellular Signal.

[16] Xu X, Wu Y, Yi K, Hu Y, Ding W, Xing C (2021). IRF1 regulates the progression of colorectal cancer via interferon–induced proteins. Int J Mol Med..

[17] Meyer K, Kwon YC, Liu S, Hagedorn CH, Ray RB, Ray R (2015). Interferon-α inducible protein 6 impairs EGFR activation by CD81 and inhibits hepatitis C virus infection. Sci Rep.

[18] Wu X, Wang S, Yu Y, Zhang J, Sun Z, Yan Y, Zhou J (2013). Subcellular proteomic analysis of human host cells infected with H3N2 swine influenza virus. Proteomics.

[19] Hussein HAM, Briestenska K, Mistrikova J, Akula SM (2018). IFITM1 expression is crucial to gammaherpesvirus infection, in vivo. Sci Rep.

[20] Cheriyath V, Kaur J, Davenport A, Khalel A, Chowdhury N, Gaddipati L (2018). G1P3 (IFI6), a mitochondrial localised antiapoptotic protein, promotes metastatic potential of breast cancer cells through mtROS. British J Cancer.

[21] Jung JJ, Jeung HC, Chung HC, Lee JO, Kim TS, Kim YT, Noh SH, Rha SY (2009). In vitro pharmacogenomic database and chemosensitivity predictive genes in gastric cancer. Genomics.

[22] Lui AJ, Geanes ES, Ogony J, Behbod F, Marquess J, Valdez K, Jewell W, Tawfik O, Lewis-Wambi J (2017). IFITM1 suppression blocks proliferation and invasion of aromatase inhibitor-resistant breast cancer in vivo by JAK/STAT-mediated induction of p21. Cancer Lett..

[23] Shirai K, Shimada T, Yoshida H, Hayakari R, Matsumiya T, Tanji K, Murakami M, Tanaka H, Imaizumi T (2017). Interferon (IFN)-induced protein 35 (IFI35) negatively regulates IFN-beta-phosphorylated STAT1-RIG-I-CXCL10/CCL5 axis in U373MG astrocytoma cells treated with polyinosinic-polycytidylic acid. Brain Res.

[24] Yang W, Tan J, Liu R, Cui X, Ma Q, Geng Y, Qiao W (2012). Interferon-γ upregulates expression of IFP35 gene in HeLa cells via interferon regulatory factor-1. PloS one.

[25] Yang XD, Xu XH, Zhang SY, Wu Y, Xing CG, Ru G, Xu HT, Cao JP (2015). Role of miR-100 in the radioresistance of colorectal cancer cells. Am J Cancer Res.

[26] Tang Z, Li C, Kang B, Gao G, Li C, Zhang Z (2017). GEPIA: a web server for cancer and normal gene expression profiling and interactive analyses. Nucleic Acids Res.

[27] Xu X, Song C, Chen Z, Yu C, Wang Y, Tang Y, Luo J (2018). Downregulation of HuR inhibits the progression of esophageal cancer through interleukin-18. Cancer Res Treatment.

[28] Hua F, Shang S, Yang Y-W, Zhang H-Z, Xu T-L, Yu J-J, Zhou D-D, Cui B, Li K, Lv X-X (2019). TRIB3 interacts With β-catenin and TCF4 to increase stem cell features of colorectal cancer stem cells and Tumorigenesis. Gastroenterology.

[29] He Y, Xu W, Xiao Y, Pan L, Chen G, Tang Y, Zhou J, Wu J, Zhu W, Zhang S (2018). Overexpression of peroxiredoxin 6 (PRDX6) promotes the aggressive phenotypes of esophageal squamous cell carcinoma. J Cancer.

[30] Xiao C, Wang Y, Zheng M, Chen J, Song G, Zhou Z, Zhou C, Sun X, Zhong L, Ding E (2018). RBBP6 increases radioresistance and serves as a therapeutic target for preoperative radiotherapy in colorectal cancer. Cancer Sci.

